# Reinfection of urogenital schistosomiasis in pre-school children in a highly endemic district in Northern Zimbabwe: a 12 months compliance study

**DOI:** 10.1186/s40249-018-0483-7

**Published:** 2018-09-21

**Authors:** Masceline Jenipher Mutsaka-Makuvaza, Zvifadzo Matsena-Zingoni, Cremance Tshuma, Sunanda Ray, Xiao-Nong Zhou, Bonnie Webster, Nicholas Midzi

**Affiliations:** 1 Department of Medical Microbiology, College of Health Sciences, University of Zimbabwe, P. O. Box A178, Avondale, Harare Zimbabwe; 2 National Institute of Health Research, Ministry of Health and Child Care, P.O. Box CY573, Causeway, Harare Zimbabwe; 3 Division of Epidemiology and Biostatistics, School of Public Health, Faculty of Health Sciences, University of Witwatersrand, 27 St Andrews’ Road, Parktown, Johannesburg, 2193 South Africa; 4 Mashonaland Central Provincial Health Office, Ministry of health and Child Care, Bindura, Zimbabwe; 5 Department of Community Medicine, College of Health Sciences, University of Zimbabwe, P. O. Box A178, Avondale, Harare Zimbabwe; 6 National Institute of Parasitic Diseases, Chinese Centre for Disease Control and Prevention, Shanghai, People’s Republic of China; 7 Parasites and Vectors Division, National History Museum, London, UK

**Keywords:** Schistosomiasis, Prevalence, Participation, Compliance, Sample submission, Pre-school aged children, Reinfection

## Abstract

**Background:**

In light of the shift to aiming for schistosomiasis elimination, the following are needed: data on reinfection patterns, participation, and sample submission adherence of all high-risk age groups to intervention strategies. This study was conducted to assess prevalence, reinfections along with consecutive participation, sample submission adherence, and effect of treatment on schistosomiasis prevalence in children aged five years and below in an endemic district in Zimbabwe, over one year.

**Methods:**

The study was conducted from February 2016–February 2017 in Madziwa area, Shamva district. Following community mobilisation, mothers brought their children aged 5 years and below for recruitment at baseline and also urine sample collection at baseline, 3, 6, 9 and 12 months follow up surveys. At each time point, urine was tested for urogenital schistosomiasis by urine filtration and children found positive received treatment. *Schistosoma haematobium* prevalence, reinfections as well as children participation, and urine sample submission at each visit were assessed at each time point for one year.

**Results:**

Of the 535 children recruited from the five communities, 169 (31.6%) participated consecutively at all survey points. The highest mean number of samples submitted was 2.9 among communities and survey points. *S. haematobium* prevalence significantly reduced from 13.3% at baseline to 2.8% at 12 months for all participants and from 24.9% at baseline to 1.8% at 12 months (*P* <  0.001) for participants coming at all- time points. Among the communities, the highest baseline prevalence was found in Chihuri for both the participants coming consecutively (38.5%, 10/26) and all participants (20.4%, 21/103). Reinfections were significantly high at 9 months follow up survey (*P* = 0.021) and in Mupfure (*P* = 0.003). New infections significantly decreased over time (*P* <  0.001). Logistic regression analysis showed that the risk of acquiring schistosomiasis was high in some communities (*P* <  0.05).

**Conclusions:**

*S. haematobium* infections and reinfections are seasonal and depend on micro-geographical settings. The risk of being infected with schistosomes in pre-school aged children increases with increasing age. Sustained treatment of infected individuals in a community reduces prevalence overtime. Participation compliance at consecutive visits and sample submission adherence are important for effective operational control interventions.

**Electronic supplementary material:**

The online version of this article (10.1186/s40249-018-0483-7) contains supplementary material, which is available to authorized users.

## Multilingual abstracts

Please see Additional file 1 for translations of the abstract into the six official working languages of the United Nations.

## Background

Schistosomiasis is a waterborne disease affecting the poorest of the poor communities causing devastating morbidities with an estimated 200 thousand death annually in the world. Recent global estimates show that 206.4 million people comprising of 111.2 million school aged children and 95.2 million adults need treatment [1, 2]. Urogenital schistosomiasis is a major public health concern in sub-Saharan Africa where approximately 436 million people are at risk of infection and 112 million people are infected with *Schistosoma haematobium* [3]. The 65.21 World Health Assembly (WHA) resolution to eliminate schistosomiasis [4], requires an all-inclusive programme for all high risk groups. Primary school aged children, children aged five years and below (pre-school aged children, PSAC) and women in endemic foci are all considered to be high risk groups but until now preventive chemotherapy is mostly targeted at primary school aged children only [2, 5, 6]. This leaves out PSAC and women who also experience severe morbidity related to schistosomiasis [7–14]. Although these studies show that PSAC are also infected with schistosomes, there is scarcity of data showing the dynamics of reinfections to inform current and future control strategies. In addition, for the success of any health programme, consistent participation (compliance) of the targeted population in programme interventions is a prerequisite [15].

In light of the epidemiological data revealing that PSAC might be a continuous reservoir of infection [6, 7, 9–12, 14], the World Health Organization (WHO) in 2010 [5] recommended treatment following observations that the current praziquantel formulation is efficacious and safe in this age group. However, WHO still recommends the exclusion of this group in mass drug administration programmes indicating that they are treated on an individual case basis, by qualified medical personnel, [5, 16] mainly because of lack of an appropriate paediatric praziquantel formulation [7, 9, 10, 17–20]. In addition, there is insufficient epidemiological data in other settings [6]. The current commercially available praziquantel tablet needs to be broken to get the correct dosage, before crushing it to avoid choking and then mixed with a sweetener to avoid the bitter taste [17]. While the current drive now is to come up with an appropriate praziquantel formulation for PSAC, it is also important to consider reinfections, compliance, and sample submission adherence of this age group to enable diagnosis before treatment.

Most PSAC in poor communities are not enrolled in early childhood development centres and thus their accessibility for participation might be problematic. The participation of PSAC cannot be derived from the current school based control programmes that, are normally delivered at schools resulting in high participation compared to community based programmes [21–25]. More so, PSAC’s participation in health programmes is depended on mothers/caregivers’ discretion depending on their perception of the programme [26–28]. In the Expanded Programme on Immunisation (EPI) in sub-Saharan Africa these children are accessed successfully through primary health care (PHC) programmes. In this current study in Zimbabwe, these young children were also accessed through the PHC [29]. Given that monitoring of reinfections is dependent on the religious participation to follow up programmes, there is a need to ascertain to what extent do PSAC comply to successive participation and if the PHC system is effective regards the accessibility of PSAC for participation in schistosomiasis control programmes.

The PSAC should be able to submit samples required for diagnosis to understand disease epidemiology [10, 12, 14, 29] which, is crucial for focal control towards schistosomiasis elimination [30–32]. Due to day-to-day variability in egg production, the analysis of multiple consecutive urine samples is essential for parasitological urogenital schistosomiasis diagnosis [33]. Collecting samples on three consecutive days in PSAC depend on the mother/caregivers’ willingness to bring their children to the sample collection point consecutively and their willingness to instruct or assist their children to provide a urine sample in a container at a particular time. Assessing how mothers respond to a schistosomiasis control program by bringing their children and the compliance of children in providing urine for three consecutive days is therefore essential.

To our knowledge there is no study that has assessed compliance, sample submission adherence, together with understanding the epidemiology of schistosomiasis in PSAC in Zimbabwe and elsewhere. This study assessed the prevalence, reinfections, compliance, sample submission adherence, new infections, and intensity of *S. haematobium* infections in PSAC at three monthly intervals for 12 months in a micro-geographical setting in an endemic district in Zimbabwe.

## Methods

### Study area

**Fig. 1:**
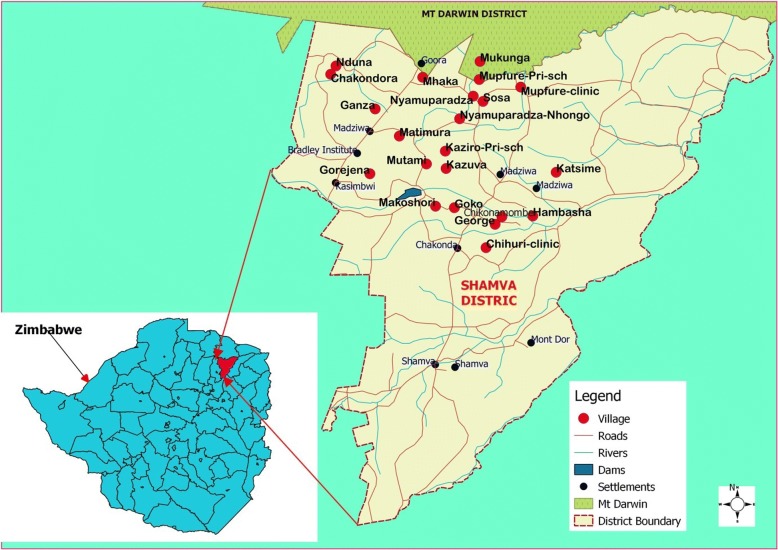
Location map for schistosomiasis study area in Madziwa area, Shamva district, Zimbabwe. The villages where the study was carried out are shown in the map

The study was conducted in five rural communities in Madziwa area, Mashonaland province, in an endemic district for both urogenital and intestinal schistosomiasis. The chosen villages (represented in Fig. 1), have an estimated population size of 4054, covering approximately 165 km^2^. The residents are primarily subsistence farmers. The maShona tribe dominate the area and most of them are apostolic sect followers who have a habit of baptizing their congregants in open water sources. The communities are served by one rural clinic and one rural health centre. There are no major water development projects in the area. The area is drained by one big river (Mupfure), and three small rivers (Nyamaruru, Nyarukunda and Kamoyo) serving as sources of water for most household activities and farming purposes. Residents go to nearby rivers for bathing, fishing, swimming, washing, to collect water for their gardens and subsistence farming. There are few boreholes that, are used by residents from up to 5 km away. Here they collect water for drinking purposes only, due to the distance from their homes.

Madziwa area has a relatively gentle terrain with fewer places being classified as steep slopes and rocky. The area has a varied soil pattern characterised by coarse grained sands, loamy sand, and occasionally sandy clay soils. The vegetation type is mainly Savanna grasslands with scattered woodlands [34]. The area receives high rainfall averaging 175 mm/month during the rainy season (November–March) but is dry between May and October. The winter season temperature ranges from 15 to 20 °C while summer temperatures range between 23 and 32 °C [34, 35].

### Study design

This was a prospective cohort study measuring the reinfections, new infections, prevalence participation to consecutive three monthly survey points (compliance), and adherence to sample submission of urogenital schistosomiasis in PSAC, using samples collected at five data collection points. This was a sub-study of the main study investigating host-schistosome interactions: Disease burden in children aged five years and below, mothers and compliance during a one year schistosomiasis control programme in a district described as highly endemic in Zimbabwe [36]. In this endemic district, Madziwa area was chosen because it has a perennial river system and its poor water resource development. These conditions predispose to schistosomiasis infection. The study was conducted from February 2016 to February 2017.

### Study population and sample size

Children aged five years and below recruited into the study were followed up at three monthly intervals over a period of one year (month 3, 6, 9, and 12). Mothers were requested to bring their children to the clinic or EPI meeting points. The required sample size was calculated to be 363 participants using Dobson’s formula as follows:

$$ n=\frac{{Z^2}_{\raisebox{1ex}{$a$}\!\left/ \!\raisebox{-1ex}{$2$}\right.} pq}{d^2} $$

Where Z is the *Z* value for the 95% confidence interval, that is alpha = 5% (*z* = 1.96)

p = proportion/prevalence of the outcome to be investigated (*p* = 0.62)

q = 1-*p* = 0.38

d = precision for the given confidence interval expected expressed as a decimal (*d* = 0.05)

*n* **=** 363

The expected prevalence of schistosomiasis observed by Midzi et al. (62%) in school-aged children [36] was used as a proxy for the PSAC. Due to the overwhelming response from the community at recruitment stage, 535 participants were recruited into the study. Surpassing the sample size of 363 took care of anticipated effect size and other hidden factors that may affect the interpretation of the results of the survey if a small sample size were used.

### Demographic data and urine collection

Demographic data including age, sex and residential location (village and community names) were collected from the caregiver of participants using a questionnaire. Each participant was given a unique identification number. The same number was used on the questionnaire, urine sample and results of the participants throughout the study. Approximately 50 ml of urine was collected into dry and clean screw capped containers from each participant. Urine was collected for three consecutive days between 10:00 am and 2:00 pm, a period when peak egg excretion is expected [33]. The caregivers were instructed on how to assist their children in collecting the sample. For young children who could not use the screw capped containers, urine bags (Hollister 7511 U-Bag Urine Specimen Collector, Hollister Inc., Chicago, IL, USA) were provided to the caregivers after being instructed on how to use them. The collected urine samples were transported to the field laboratory set up at a nearby rural health centre for processing and examination within 2 hours of collection.

### Laboratory analysis

Parasitological diagnosis of urogenital schistosomiasis was performed using the urine filtration technique [37] at baseline and then at 3 months, 6 months, 9 months and 12 months follow up points. One urine filtration was performed on a single urine sample every day for 3 days at each survey point. A thoroughly mixed 10 ml urine specimen was filtered through a 12 μm pore sized nitrile filter membrane and transferred onto a microscope slide labeled with the participant’s identification number. After addition of 10% iodine, the slides were examined for *S. haematobium* eggs under the laboratory compound microscope (Nikon, UK) using the 10× objective lens. *S. haematobium* eggs were morphologically identified by their oval shape and characteristic terminal spine [38].

For each child found positive for *S. haematobium* the intensity of infection was expressed as the number of eggs per 10 ml urine. For urine samples less than 10 ml, the volumes were measured before filtration and the egg count adjusted proportionally.

### Treatment

All participants found positive for urogenital schistosomiasis were entered into a treatment register and given a single dose of praziquantel at the recommended dose of 40 mg/kg body weight. The praziquantel dose was administered by the local clinic nurse, assisted by the child’s mother. For children that could not swallow the tablets, the praziquantel was crushed and mixed with orange juice to help with ingestion. Only children found positive for urogenital schistosomiasis using the parasitology methods were given treatment and this was recorded in the treatment register at each survey point.

### Compliance assessment

Children were assessed for their compliance in participation at each consecutive survey point and adherence in submission of urine specimens for three successive days as a requirement for the diagnostic procedure.

### Data analysis

Data analysis was performed using STATA 15.2 (Stata Corp, Collage station, TX, USA). Age was categorized into three groups (0–1, 2–3 and 4–5 years). Descriptive analysis was used to describe the age, sex, reinfections, new infections, prevalence, compliance and adherence to sample submission at different time points in each community. The proportion test was used to test the significant difference in prevalence of urogenital schistosomiasis in communities at the different survey points, age groups, and sexes at 95% confidence level.

Due to logistical reasons drug efficacy was not measured at 6 weeks so it could not be ascertained whether those who were positive at baseline and were also found positive at 3 months had been reinfected or had not cleared infections. The reinfection rates in the PSAC were calculated from 6 months to 12 months as follows:

$$ \mathrm{Proportions}\ \mathrm{of}\ \mathrm{reinfections}\ \mathrm{per}\ \mathrm{t}\mathrm{ime}\ \mathrm{point}=\left(\mathrm{Number}\ \mathrm{of}\ \mathrm{participants}\ \mathrm{with}\ \mathrm{reinfections}\ \mathrm{a}\mathrm{t}\ \mathrm{a}\ \mathrm{survey}\ \mathrm{point}/\mathrm{Number}\ \mathrm{of}\ \mathrm{participants}\ \mathrm{with}\ \mathrm{infections}\ \mathrm{a}\mathrm{t}\ \mathrm{t}\mathrm{hat}\ \mathrm{particular}\ \mathrm{time}\ \mathrm{point}\right)\times 100 $$

Infection intensity was categorized according to WHO guidelines (light intensity: 1–49 eggs/10 ml, heavy intensity: ≥ 50 eggs/10 ml) [2] and compared at different time points. Arithmetic mean infection intensity was calculated by adding the number of eggs observed per day divided by the number of days of urine collection. This was expressed as the number of eggs per 10 ml of urine examined. The arithmetic mean egg intensity was calculated for all participants investigated.

In order to demonstrate the effect of praziquantel treatment on prevalence and intensity of infection, the prevalence and mean egg intensity reduction rates at baseline and at 12 months was calculated as follows:

$$ \mathrm{Prevalence}\ \mathrm{reduction}\ \mathrm{rate}=\left(\mathrm{prevalence}\ \mathrm{at}\ \mathrm{baseline}\ \mathrm{before}\ \mathrm{treatment}-\mathrm{prevalence}\ \mathrm{at}\ 12\ \mathrm{months}\ \mathrm{follow}\ \mathrm{up}\ \mathrm{point}\right)/\left(\mathrm{prevalence}\ \mathrm{at}\ \mathrm{baseline}\ \mathrm{before}\ \mathrm{treatment}\right)\times 100 $$

$$ \mathrm{Egg}\ \mathrm{reduction}\ \mathrm{rate}=\left(\mathrm{mean}\ \mathrm{eggs}/10\ \mathrm{ml}\ \mathrm{urine}\ \mathrm{at}\ \mathrm{baseline}\ \mathrm{before}\ \mathrm{treatment}-\mathrm{mean}\ \mathrm{eggs}/10\ \mathrm{ml}\ \mathrm{at}\ 12\ \mathrm{months}\ \mathrm{follow}\ \mathrm{up}\ \mathrm{point}\right)/\left(\mathrm{mean}\ \mathrm{eggs}/10\ \mathrm{ml}\ \mathrm{urine}\ \mathrm{at}\ \mathrm{baseline}\ \mathrm{before}\ \mathrm{treatment}\right)\times 100. $$

The cure rate was calculated as:

$$ \left[\mathrm{the}\ \mathrm{number}\ \mathrm{of}\ \mathrm{participants}\ \mathrm{excreting}\ \mathrm{no}\;S. haematobium\ eggs/\mathrm{the}\ \mathrm{number}\ \mathrm{of}\ \mathrm{participants}\ \mathrm{with}\ \mathrm{confirmed}\ \mathrm{infection}\ \mathrm{at}\ \mathrm{baseline}\right]\times 100 $$

Univariate logistic regression model was used to identify factors associated with *S. haematobium* infection. Multivariate logistic regression was fitted to control the possible effect of confounders. The variables having independent association with *S. haematobium* infection were identified based on odds ratio with 95% confidence interval and *P*-values less than 0.05.

## Results

Overall, 535 participants (291 males and 244 females) from five communities were recruited into the study at baseline. The total number of children recruited, their gender and age distribution at baseline stratified by community are shown in Table 1. Among the age groups, the highest recruitment was of the 2–3 year old (46.9%).

**Table 1:** Overall demographic characteristics at baseline and subsequent follow up-surveys of pre-school aged children in five communities in Madziwa area, Shamva district, Zimbabwe

Variable	Total *n =* 535	Chakondora *n =* 138	Chihuri *n =* 103	Kaziro *n =* 67	Mupfure *n =* 169	Nduna *n =* 58
Baseline survey	*n* (%)	*n* (%)	*n* (%)	*n* (%)	*n* (%)	*n* (%)
Sex						
Male	291(54.4)	71 (51.5)	59 (57.3)	40 (59.7)	92 (54.4)	29 (50)
Female	244(45.6)	67 (48.5)	44 (42.7)	27 (40.3)	77 (45.6)	29 (50)
Age group (years)						
0*–*1	83 (15.5)	30 (21.7)	9 (8.7)	8 (11.9)	23 (13.6)	13 (22.4)
2*–*3	251 (46.9)	67 (48.6)	51 (49.5)	32 (47.8)	79 (46.8)	22 (37.9)
4*–*5	201 (37.6)	41 (29.7)	43 (41.8)	27 (40.3)	67 (39.6)	23 (39.7)
Participation at each visit*						
Baseline	535 (100)	138 (100)	103 (100)	67 (100)	169 (100)	58 (100)
3 months	289 (54.0)	70 (50.7)	48 (46.6)	24 (35.8)	109 (64.5)	38 (65.5)
6 months	262 (49)	65 (47.1)	44 (42.7)	22 (32.8)	97 (57.4)	34 958.6)
9 months	289 (54)	65 (47.1)	52 (50.5)	41 (61.2)	110 (65.1)	21 (36)
12 months	390 (72.9)	90 (65.2)	67 (65.1)	52 (77.6)	141 (83.4)	40 (69)
Compliant group^a^	169 (31.6)	41 (29.7)	26 (25.2)	12 (17.9)	72 (42.6)	18 (31.0)
Mean number of Urine specimens^b^						
Baseline	2.4	2.1	2.4	2.3	2.6	2.4
3 months	2.2	1.8	2.5	2.3	2.3	2.2
6 months	2.6	2.5	2.7	2.8	2.8	2.4
9 months	2.8	2.8	2.8	2.9	2.8	2.9
12 months	2.6	2.4	2.7	2.7	2.7	2.5

*Children present at a survey regardless of consistency. The percentage of children at each follow up visit was calculated as a percentage of the number of participants recruited at baseline survey

^a^Participants who came successively at all survey points

^b^Mean number of urine specimens collected per participant at baseline and follow up surveys for all participants at each follow up visit is shown

### Participation and adherence to submission of samples for the PSAC

**Fig. 2:**
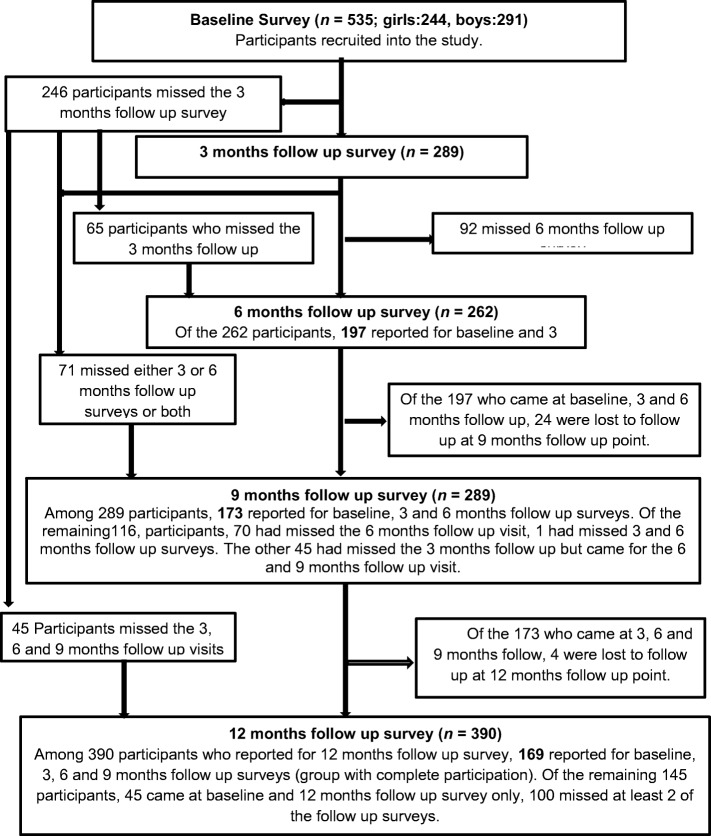
Description of participation characteristics of participants to follow up visits. Participants recruited at baseline were monitored for their participation at follow up visits. Those who were consistent at consecutive participation were recorded. Absenteeism at follow up visits was also recorded

The participation of PSAC at consecutive survey points is described in Table 1 and Fig. 2. The dynamics of participation are elaborated in Fig. 2. While 390 (72.9%) participants finally turned up at 12 months, those compliant were only 169 (31.6%). It varied among communities from 17.9% (Kaziro) to 42.6% (Mupfure). The overall mean of urine samples collected per participant at each time point varied from 1.8 to 2.9 among communities and survey points.

### *Schistosoma haematobium* infection, intensity, and reduction over time

**Fig. 3:**
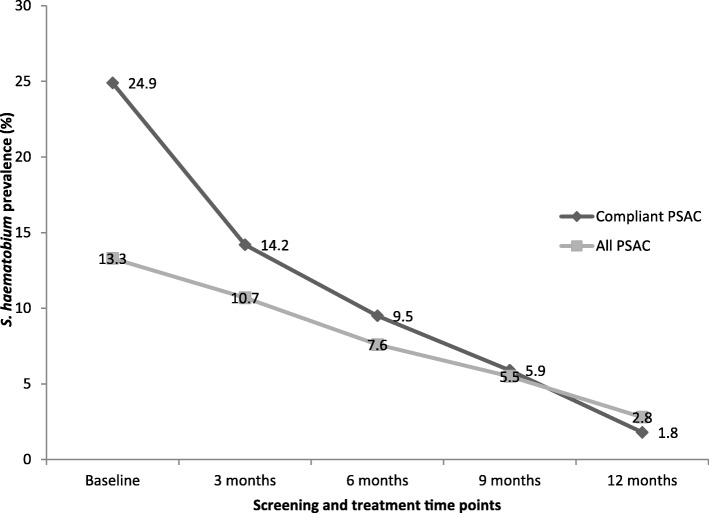
Overall prevalence of *S. haematobium* infection in all pre-school aged children and compliant pre-school aged children over time. The *S. haematobium* prevalence for all the participants versus only those who came successively (compliant) at each time point is shown

**Fig. 4:**
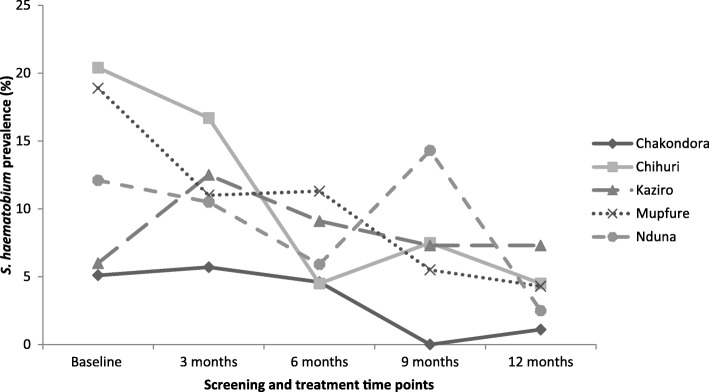
Prevalence of *S. haematobium* infection in all pre-school aged children attending at each time point by community. The *S. haematobium* prevalence for all the participants regardless of their consistency in participation at all time points is shown for each community

**Fig. 5:**
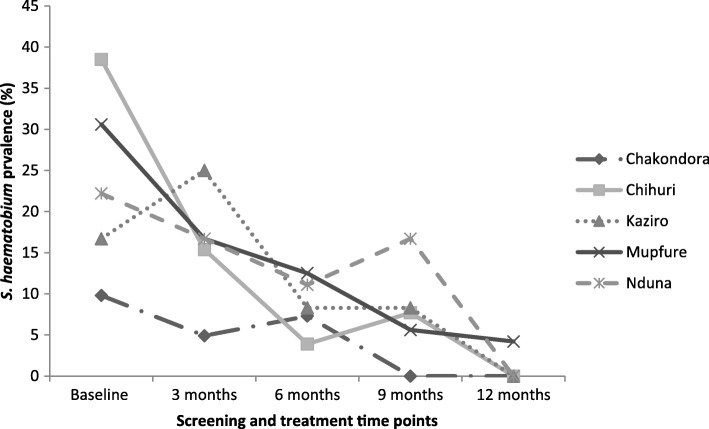
Prevalence of *S. haematobium* infection in compliant pre-school aged children attending at each time point by community. The *S. haematobium* prevalence for each community for those participants who came successively at each time point is shown

The overall *S. haematobium* prevalence in all participants declined consistently from 13.3% (*95% CI*: 10.5–16.4) at baseline, 10.7% (95% *CI*: 7.4–14.9) at 3 months, 7.6% (95% *CI*: 4.7–11.5) at 6 months, 5.5% (95% *CI*: 2.2–8.8) at 9 months to 2.8% (95% *CI*: 1.4–5.0) at 12 months respectively. The overall *S. haematobium* prevalence in compliant participants consistently declined from 24.9% (95% *CI*: 18.5–32.1) at baseline, 14.2% (95% *CI*: 9.3–20.4) at 3 months, 9.5% (95% *CI*: 5.5–14.9) at 6 months, 5.9% (95% *CI*: 2.9–10.6) at 9 months to 1.8% (95% *CI*: 0.4–5.1) at 12 months (Fig. 3). Overall, the prevalence was significantly higher in the compliant group than in all participants (*P* = 0.031) at baseline whilst the difference was insignificant at all follow–up points (*P* > 0.05). The prevalence of *S. haematobium* significantly declined from baseline to 12 months by 78.9% in all PSAC and 92.8% in the compliant PSAC (*P* <  0.001) (Tables 2 and 3). Among the communities, baseline prevalence was generally higher in the compliant group compared to all PSAC (Figs. 4 and 5). Chakondora had the least trend of *S. haematobium* infection over time for both all participants and the compliant group. Chihuri had the highest prevalence at baseline for both the compliant group (38.5%) and all participants (20.4%). Chihuri, Kaziro, Mupfure, and Nduna showed fluctuating patterns of prevalence over time starting from 38.5, 16.7, 30.6, and 22.2%, for the compliant PSAC, and from 20.4, 6.0, 18.9 and 12.1% for all participants respectively (Figs. 4 and 5). In the compliant group, prevalence was highest in Kaziro at 3 months (25%), Mupfure at 6 months (12.5%), Nduna at 9 months (16.7%), whilst at 12 months infection was only found in Mupfure at 4.2% implying 100% infection reduction for the other communities (Fig. 5 and Table 3). Considering all PSAC, only Kaziro achieved a 100% reduction (Fig. 4 and Table 2). However, the decline in prevalence in all communities was significant for all participants and the compliant group (*P* <  0.05).

**Table 2:** Changes in prevalence and intensity of *S. haematobium* infection among all pre-school aged children participants

Variable	Number of children examined		Prevalence			Mean egg count		
			*n* % (95% *CI*)		% Reduction	Eggs/10 ml urine (95% *CI*)		% Reduction
	Baseline	12 months	Baseline	12 months		Baseline	12 months	
Overall	535	390	71 13.3 (10.5–16.4)	11 2.8(1.4–5.0)	78.9	2.1 (0.8–3.3)	0.2 (0–0.4)	90.5
Community								
Chakondora	138	90	7 5.1 (2.1–10.2)	1 1.1(0–6.0)	78.4	0.2 (0–0.4)	0.03 (0–0.1)	85.0
Chihuri	103	67	21 20.4 (13.1–29.5)	3 4.5(0.9–12.5)	77.9	4.6 (−1.3–10.6)	0.9 (0–2.0)	80.4
Kaziro	67	52	4 6.0 (1.7–14.6)	0	100	2.2 (−0.2–4.6)	0	100
Mupfure	169	141	32 18.9 (13.3–25.7)	6 4.3(1.6–9.0)	77.2	2.7 (1.1–4.2)	0.1 (0–0.3)	96.3
Nduna	58	40	7 12.1 (5.0–23.3)	1 2.5(0.1–13.2)	79.3	0.2 (0–0.4)	0.1 (− 0.1–0.4)	50.0
Age (years)								
0–1	83	55	4 4.8 (1.3–11.9)	0	100	0.4 (0–1.0)	0	100
2–3	251	185	27 10.8 (7.2–15.3)	5 2.7(0.9–6.2)	75.0	1.9 (−0.5–44.4)	0.2 (0–0.4)	89.5
4–5	201	150	40 19.9 (14.6–26.1)	6 4.0(1.5–8.5)	79.9	2.9 (1.4–4.4)	0.4 (0–0.8)	86.2
Sex								
Male	291	218	36 12.4 (8.8–16.7)	6 2.8(1.0–5.9)	77.4	1.9 (0.9–2.8)	0.3 (−0.1–0.6)	84.2
Female	244	172	35 14.3(10.2–19.4)	5 2.9(1.0–6.7)	79.7	2.3 (0–4.9)	0.2 (0–0.4)	91.3

The change in prevalence and intensity calculated from baseline and 12 months follow up for all participants regardless of successive participation

**Table 3:** Changes in Prevalence and intensity of *S. haematobium* infection among the compliant pre-school aged children/group

Variable	Number of children examined	Prevalence			Mean egg count		
		Number of infected individuals % (95% *CI*)		% Reduction	Eggs/10 ml urine (95% *CI*)		% Reduction
		Baseline	12 months		Baseline	12 months	
Overall	169	42 24.9 (18.5–32.1)	3 1.8 (0.4–5.1)	92.8	3.1 (1.5–4.8)	0.1(0–0.1)	96.8
Community							
Chakondora	41	4 9.8 (2.7–23.1)	0	100	0.4 (−0.2–1.1)	0	100
Chihuri	26	10 38.5 (20.2–59.4)	0	100	3.8 (0.8–6.7)	0	100
Kaziro	12	2 16.7(2.1–48.4)	0	100	4.8 (−4.5–14.0)	0	100
Mupfure	72	22 30.6 (20.2–42.5)	3. 4.2 (0.9–11.7)	86.3	4.8 (1.4–8.2	0.1(−0.04–0.3)	97.9
Nduna	18	4 22.2(6.4–47.6)	0	100	0.4 (−0.2–1.0)	0	100
Age (years)							
0–1	1	1 100	0	100	9.7	0	100
2–3	84	14 16.7(9.4–26.4)	1 1.2 (0–6.5)	92.8	0.9(0.2–1.6)	0.1 (0–0.2)	88.9
4–5	84	27 32.1(22.4–43.2)	2 2.4 (0.3–8.3)	92.5	5.2(2.1–8.4)	0.04 (0–0.1	99.2
Sex							
Male	89	20 22.5(14.3–32.6)	1 1.1(0–6.1)	95.1	3.8(1.0–6.6)	0.01(0–0.04)	99.7
Female	80	22 27.5(18.1–38.6)	2 2.5(0.3–8.7)	90.9	2.4(0.8–3.9)	0.1(0–0.2)	95.8

The change in prevalence and intensity calculated from baseline and 12 months follow up for all compliant participants

In the compliant group, there was a significant reduction of prevalence of *S. haematobium* infection by sex from baseline to 12 months follow-up survey: 95.1% for males (*P* = 0.016) and 90.9% for females (*P* = 0.005). However, the difference in reduction between sexes was not significant (*P* = 0.6056). In the present study, there was a general increase in *S. haematobium* prevalence with increasing age from 16.7 and 1.2% in 2–3 year old group to 32.1% and 2.4% in 4–5 year olds at both baseline and 12 months respectively. The only 0–1 year old participant who was compliant had *S. haematobium* infection at baseline, which was successfully treated. There was a significant reduction of *S. haematobium* infection with age from baseline to 12 months for all the age groups in both the compliant group (*P* <  0.001, Table 3) and for all participants (*P* <  0.05, Table 2). The overall mean egg count for the compliant participants declined by 96.8% from baseline to the 12 months follow-up survey in the compliant group (Table 3) and 90.5% for all the participants (Table 2). Among sex strata, mean egg intensity reduction was above 95% in the compliant group (Table 3) and 84% for all participants respectively (Table 2). Table 4 shows the dynamics of infection among groups with different attendances. Although this was not significant (*P* = 0.229), those who did not attend all rounds, had higher prevalence at 12 months compared to compliant PSAC.

**Table 4:** Overall description of attendance and prevalence dynamics of participants at baseline and follow up points

Attendance at Survey points	Baseline		3 months		6 months		9 months		12 months	
	*n*	*n* (%)	*N*	*n* (%)	*n*	*n* (%)	*n*	*n* (%)	*n*	*n* (%)
		95% *CI*		95% *CI*		95% *CI*		95% *CI*		95% *CI*
1	169	42(24.9)	169	24(14.2)	169	16(9.5)	169	10(5.9)	169	3(1.8)
		18.5–32.1		9.3–20.4		5.5–14.9		2.9–10.6		0.4–5.1
2	100	4 (4.0)	0	–	0	–	0	–	0	–
		1.1–9.9								
3	11	2(18.2)	11	1 (9.1)	0	–	0	–	0	–
		2.3–51.8		0.2–41.3						
4	6	2 (33.2)	6	0	6	0	0	–	0	–
		4.3–77.7		–						
5	4	1 (25)	4	1 (25)	4	0	4	0	0	–
		0.6–80.6		0.6–80.6		–				
6	18	2(11.1)	18	1(5.6)	18	0	0	–	18	1(5.6)
		1.4–34.7		0.1–27.3		–				0.1–27.3
7	34	8(23.5)	34	3(8.8)	0	–	34	1(2.9)	34	1(2.9)
		10.7–41.2		1.9–23.7				0–15.3		0.1–15.3
8	1	0	1	0	0	–	1	0	0	–
		–		–						
9	46	2(4.3)	46	1(2.2)	0	–	0	–	46	–
		0.5–14.8		0.1–11.5						
10	11	0	0	–	11	0	0	–	0	–
		–				–				
11	4	1(25)	0	–	4	0	4	1 (25)	0	–
		0.6–80.6				–		0.6–80.6		
12	41	2(4.9)	0	–	41	4 (9.8)	41	1(2.4)	41	1(2.4)
		0.6–16.5				2.7–23.1		0–12.9		0.1–12.9
13	9	0	0	–	9	0	0	–	9	0
14	8	0	0	–	0	–	8	0	0	–
15	28	3(10.7)	0	–	0	–	28	3(10.7)	28	2 (7.1)
		2.3–28.2						2.3–28.2		0.9–23.5
16	45	2 (4.4)	0	–	0	–	0	–	45	3 (6.7)
		0.5–15.1								1.4–18.3

*N*: the number of participants examined at a survey point for each group; *n*(%) is the number and percentage who were *S. haematobium* positive at each survey point for each group

1: All survey points; 2: Baseline only; 3: Baseline and 3 months only; 4: Baseline, 3 months, and 6 months only; 5: Baseline, 3 months, 6 months and 9 months only; 6: Baseline, 3 months, 6 months and 12 months; 7: Baseline, 3 months, 9 months and 12 months; 8: Baseline, 3 months and 9 months only; 9: Baseline, 3 months, and 12 months; 10: Baseline, and 6 months only; 11: Baseline, 6 months and 9 months only; 12: Baseline, 6 months, 9 months and 12 months only; 13: Baseline, 6 months and 12 months only; 14: Baseline, 9 months only; 15: Baseline, 9 months and 12 months only; 16: Baseline, and 12 months only

### Urogenital schistosomiasis reinfections and new infections in PSAC

**Fig. 6:**
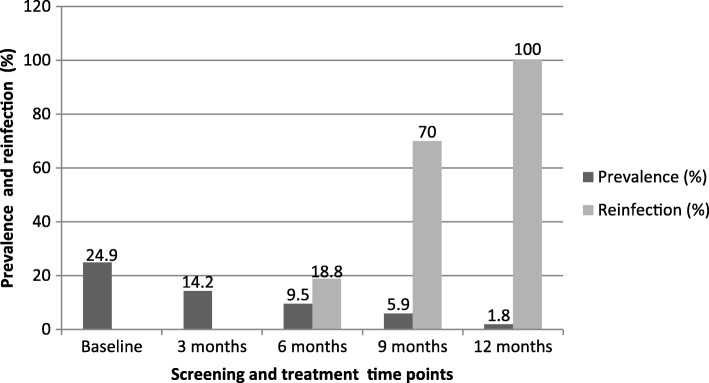
Prevalence and reinfections of *S. haematobium* in compliant pre-school aged children attending at each time. Reinfections were considered only in those who came successively (the compliant group) at each survey point. The reinfections rates were calculated as a proportion of the number of infected participants (prevalence) at a time point. Reinfection rates could not be measured at three months follow up survey because we had no 6 weeks post treatment survey to ascertain infection clearance

The reinfections at each point in time were only considered for the six, nine, and 12 months follow up survey (Fig. 6). Due to logistical reasons, there was no 6 weeks praziquantel efficacy study. Thus, we could not report if those who continuously excreted eggs at consecutive survey points as reinfections or persistent infections. Of the 71 participants found infected at baseline 42 were compliant. Of these, 13 were re-infected at the end of the 12 months; three at 6 months (August), seven at 9 months (November) and three at 12 months (February). Among the infections, the percentages of reinfections were increasing overtime. All the infections reported at 12 months were reinfections (Fig. 6). The chances of being re-infected was associated with the 9 months follow up point which falls in November (*P* = 0.021). Reinfections were also associated with community (*P* = 0.003) and distributed as follows: eight in Mupfure, four in Nduna, one in Chihuri and none in Chakondora and Kaziro.

There were 27 new infections by the end of 12 months; 18 cases at 3 months, 8 cases at 6 months and 1 case at 9 months, showing a decrease of new infection over time (*P* <  0.001). Among the communities, new infections were as follows; 13 in Mupfure, five in Chihuri, four in Nduna, three in Chakondora and two in Kaziro (*P* = 0.328).

### Risk factor analysis for *S. haematobium* infection in PSAC

Among the compliant participants, univariate logistic regression analysis showed that the risk of acquiring schistosomiasis was associated with community of origin only. In this age group (5 years and below), odds ratios could not be adjusted since age and sex of the participants were not shown to be risk factors in acquiring urogenital schistosomiasis (Table 5). Among the communities only Chihuri, Mupfure and Nduna were significantly associated with urogenital schistosomiasis. Univariate analysis showed that the risk of acquiring schistosomiasis infection was highest in Chihuri followed by Mupfure and least in Nduna (Table 5).

**Table 5:** Association between *S. haematobium* infection and community, age and sex of the compliant PSAC

Variable	Category	Univariate analysis		
		*OR*	95% *CI*	*P*-value
Community	Chakondora	1 (Reference)		
	Chihuri	8.4	2.5*–*28.2	0.001
	Kaziro	3.6	0.8*–*16.5	0.099
	Mupfure	5.8	2.0*–*16.4	0.001
	Nduna	4.6	1.2*–*17.4	0.025
Age (years)	0*–*1	1 (Reference)		
	2*–*3	6.4	*–*	0.985
	4*–*5	1.4	*–*	0.986
Sex	Male	1 (Reference)		
	Female	1.2	0.6*–*2.2	0.598

-: Not applicable

Concerning all participants, both univariate and multivariate logistic regression analysis showed that among the communities only Chihuri, Mupfure and Nduna were significantly associated with urogenital schistosomiasis. Univariate analysis showed that the risk of acquiring urogenital schistosomiasis infection was highest in Chihuri followed by Mupfure and lastly Nduna (Table 6). Multivariate logistic regression analysis after controlling the possible confounders showed that the risk of acquiring schistosomiasis infection was still highest in Chihuri followed by Mupfure and lastly Nduna. Both univariate and multivariate logistic regression analysis showed that the risk of acquiring urogenital schistosomiasis increases with increasing age. The risk of acquiring urogenital schistosomiasis in these PSAC was not associated with gender (Table 6).

**Table 6:** Association of *S. haematobium* infection in relation to community, age and sex of all PSAC

Variable	Category	Univariate analysis			Multivariate analysis		
		*OR*	95% *CI*	*P*-value	Adjusted *OR*	95% *CI*	*P*-value
Community	Chakondora	1 (Reference)			1 (Reference)		
	Chihuri	5.8	2.7*–*12.4	< 0.001	5.1	2.3*–*11.0	< 0.001
	Kaziro	1.7	0.7*–*4.6	0.27	1.5	0.6*–*4.1	0.408
	Mupfure	4.9	2.4*–*10.2	< 0.001	4.5	2.2*–*9.5	< 0.001
	Nduna	3.0	1.2*–*7.5	0.019	2.9	1.1*–*7.4	0.025
Age (years)	0–1	1 (Reference)			1 (Reference)		
	2–3	4.4	1.5*–*12.7	0.006	4.0	1.4*–*11.7	0.010
	4–5	8.0	2.8*–*22.9	< 0.001	7.1	2.5*–*20.5	< 0.001
Sex	Male	1 (Reference)					
	Female	0.9	0.6*–*1.4	0.786			

### Praziquantel treatment and cure rate

Among those infected, 100% treatment compliance was achieved at all survey points. Among the 42 compliant PSAC who were *S. haematobium* positive at baseline, the cure rate was as follows: 85.7% (36/42), 88.1% (37/42), 85.7% (36/42), and 92.9% (39/42) at three, six, nine, and 12 months follow up respectively. Considering all the PSAC who were *S. haematobium* positive at baseline, 71 (100%), 59 (83.1%), 50 (70.4%), 57 (80.3%) and 61 (85.9%) were present at three, six, nine, 12 months follow up visit. Among these, the cure rate was as follows: 83.1% (49/59), 90% (45/50), 89.5% (51/57) and 95.1% (58/61) at 3, 6, 9, and 12 months follow up respectively.

## Discussion

In the current study, compliance to consecutive participation was below 50% while the highest mean number of samples submitted at all survey points was 2.9. For the compliant PSAC, all but one community had 100% prevalence reduction from baseline to 12 months. However, for all participants, disregarding compliance participation, only one community achieved a 100% prevalence reduction. Among the follow up points, reinfections were significantly high at 9 months follow up survey. Among the communities, reinfections were significantly high in Mupfure. New infections significantly decreased over time. Overall, the mean egg count declined over time.

In this study, whilst children were followed up over 1 year, their participation in the project interventions at each follow up survey point was dependent on their mothers’/caregivers’ willingness to bring them to the sample collection and treatment points. In the survey context, women seek the approval of their husbands or male heads of the family to be able to participate in any programmes.

This is the second schistosomiasis study of PSAC in Zimbabwe but the first study in which compliance and adherence to sample submission of PSAC to a schistosomiasis control programme has been assessed. The first study evaluated immunological, parasitological diagnostic methods and morbidity associated with schistosomiasis in these young children [39–41]. Although schistosomiasis is a well-known problem in Zimbabwe, most studies [42–44] and the current national schistosomiasis control program only targets school-aged children [36]. Thus, it is well-recognised as a problem of school aged children. The assessment of reinfections in young children compliance and adherence to sample submission provides program planners some insights into the requirements of community participation in schistosomiasis control programs. When communities show a relatively low compliance, health promotion programs will need to be informed by research on community knowledge, practices and perceptions regarding schistosomiasis and its control although this study did not explore this aspect.

In this program 169 (31.6%) participated consecutively (complied) at all surveys from baseline to 12 months post treatment follow up survey (Table 1, Fig. 2). Individuals who are lost at follow up may remain as reservoirs of infection, thus sustaining schistosomiasis transmission in an area. The failure of diagnosis in these individuals may also lead to unreliable epidemiological data such as poor assessment of reinfections and new infections. Lack of an appropriate paediatric formulation and insufficient epidemiological data has contributed to exclusion of PSAC and adult populations in mass praziquantel treatment [6].

Lack of adherence to submission of urine samples for multiple consecutive days might poorly represent actual prevalence due to false negative diagnosis [33]. Our results (Table 1) suggest that most children managed to submit at least two samples per survey point.

Our findings show that with support from parents/caregivers it is possible to collect a urine sample from the PSAC on successive days demonstrating feasibility of using the egg count techniques in this age group to estimate prevalence. Diagnosis by egg detection that relies on collection of urine and or stool specimens from participants are non-invasive, inexpensive and require low resources, however, the low sensitivity of these methods is a problem especially in individuals with light infections [6].

Comparison of prevalence results of the compliant PSAC versus all PSAC (Fig. 3), indicate that the former had a significantly higher prevalence at baseline and unlike the latter group, showed a steep decline to 1.8% prevalence at 12 months giving a higher prevalence reduction of 92%. Likewise, when compared to all PSAC (Table 2), it became apparent that the compliant group (Table 3) had a significantly higher mean egg count at baseline and unlike the former group, showed a steep decline 0.1 (0*–*0.1) mean egg count at 12 months giving a higher mean egg count reduction of 96.8%. These findings demonstrate that usually commitment to a follow up programme requirements is influenced by infection status and associated symptoms at the start of the programme. Those who are infected realise the benefits of the programme faster, thus, resulting in commitment to consecutive follow up surveys.

The study findings indicate that all but one community achieved zero prevalence at 12 months (considering the complaint group) while only one community had zero prevalence when all participants regardless of compliance were included [Tables 2 and 3, Figs. 4 and 5]. This trend shows the importance of compliance to programme requirements as a prerequisite for the success of control interventions [15].

The significant reduction in prevalence and intensity of *S. haematobium* infection from baseline to 12 months (Tables 2 and 3) indicate that long rather than short term efforts at regular intervals are required for effective control and elimination of schistosomiasis. This is also supported by the fluctuating prevalence in communities before generally decreasing with time (Figs. 4 and 5). In addition, although it was not significant, Table 4 shows that those who were not compliant to participation had higher prevalence at 12 months compared to the compliant PSAC. Generally, given that the area has a perennial river system, the risk of disease transmission is all year round. Thus, for such an area, treatment at short time intervals is likely to suppress the disease. Thus, the current annual mass drug administration requires an upward revision emphasising multiple treatment per year than the currently on going less effective annual single round MDA. A similar observation was made in other studies elsewhere [45]. The mean egg intensity from baseline to 12 months generally indicated the presence of more light infections compared to heavy infections. This trend is generally expected in young children because of relatively less water contact exposure compared to the age group 6*–*15 years (high risk groups). Also their water contact activities are dependent on their mothers’ contact activities and whether mothers/care givers consider it safe to place their young children in shallow areas in the river/streams to play as they carry out their domestic chores. Mothers might also prefer bathing their children at home in which case they would not be accompanied by their children to the contaminated water sources. The difference in prevalence and intensity of infection, among the communities also indicates different water contact patterns. Similar observations were made in Côte d’Ivoire and Central Sudan [45, 46].

Corroborating the findings observed in Côte d’Ivoire [45], the current study has shown that the risk of being re-infected and/or acquiring new infections differed by community and survey point (Tables 5 and 6). The study also indicates that most infections observed at 9 months and all infections at 12 months follow up points were reinfections (Fig. 6). Considering that our 6 months follow up survey was carried out in August, we can easily deduce that reinfection may have occurred sometime in September–November which, agrees well with the peak transmission period in Zimbabwe [47]. All the reinfections observed at 12 months were from Mupfure community. This also confirms the focality of schistosomiasis even at a micro-geographical level and that the risk of reinfections or acquiring new infections depends on the transmission force at a particular point in time. During the dry and hot season, intermediate host snails and cercariae are concentrated on permanent water sources, thus increasing the risk of infection. The temperatures experienced during this period are also conducive for cercarial shedding [47, 48]. This is supported by our personal observations in our malacological survey within these communities (data to be published elsewhere). Logistic regression analysis (Tables 5 and 6) also support the focality of disease transmission which mostly results in reinfections and the risk of being reinfected increasing with increasing age.

Although the cure rate was generally high in this study, the results are biased due to reinfections detected at six, nine and 12 months follow up. For example, the cure rate at 12 months is not a real reflection of praziquantel efficacy given that the three positive cases were negative at 9 months. Likewise, the six participants who were positive at 9 months follow up were all reinfections. Our study design is not suitable for measuring cure rates given that we had no 4–6 weeks post treatment surveys as has been done in previous studies [49–51].

While the high-risk districts have already been mapped in Zimbabwe [36], the current study gives an insight into differences in schistosomiasis micro-epidemiology within a community and seasonality of the disease. The five communities under study were very close to each other but with different patterns of schistosomiasis infection. Therefore, for elimination of schistosomiasis, the next step in Zimbabwe and elsewhere should be determining disease epidemiology in micro-geographical settings. This will determine disease focality coupled with generating baseline data for other high risk groups in preparation for integrated schistosomiasis control strategies desirable for elimination of the disease. The current study also confirms that the risk of being infected with schistosomes in PSAC increases with increasing age as children become more independent [14].

## Conclusions

Reinfections, participation and sample submission adherence dynamics within communities should be considered before implementation of large-scale schistosomiasis control interventions since they can derail the efforts being made towards schistosomiasis elimination.

A considerable percentage of PSAC were found to have evidence of *S. haematobium* infections that could be detrimental to their growth. Observation of schistosomiasis infection in this segment of the population that is out of treatment policy can only explain a sustained transmission of the disease in the communities since they can act as reservoirs of infection until they can be included in the ongoing preventive chemotherapy.

Our study has shown that different communities within a district present different prevalence levels of schistosomiasis demonstrating existence of different epidemiological factors. Operational control strategies should consider such dynamics before implementing large scale programmes.

## Additional file

Additional file 1: Multilingual abstracts in the six official working languages of the United Nations. (PDF 728 kb)

## Abbreviations

EPI: Expanded Programme on Immunisation
MDA: Mass drug administration
PHC: Primary health care
PSAC: Pre-school aged children
WHO: World Health Organization
