# Supplementary material for: Reinfection of urogenital schistosomiasis in pre-school children in a highly endemic district in Northern Zimbabwe: a 12 months compliance study
Source: Infect Dis Poverty. 2018 Sep 21;7:102. doi: 10.1186/s40249-018-0483-7 (PMC6162945; doi:10.1186/s40249-018-0483-7)
Supplement: Supplementary file 1 — Multilingual abstracts in the six official working languages of the United Nations. (PDF 728 kb) [file 40249_2018_483_MOESM1_ESM.pdf]

Translation of the abstract into the six official working languages of the United Nations

إصابة أطفال ما قبل المدرسة بداء البلهارسيا البولية التناسلية بشكل متفشي في منطقة بشمال زمبابوي

ماشيلين جينيفر موتساكا-ماكوفازا، زيفادزو ماتسينا-زينجوني، كريمانش تشوما، صناندا راي، تسياو-نونج هاو، بوني وبستر، نيكولاس ميدزي

الملخص

الخلفية:في ضوء التحول الذي يهدف للقضاء على داء البلهارسيا, فإن المطلوب هو التالي: بيانات عن أنماط الاصابة, المشاركة والإلتزام بتقديم عينة لكل الفئات العمرية المعرضة للخطر من أجل استراتيجيات التدخل.أجريت هذه الدراسة لتقييم الانتشار والإصابات جنباً إلى جنب مع المشاركة المتتالية والإلتزام بتقديم عينة ، وتأثير العلاج على انتشار البلهارسيا في الأطفال الذين تتراوح أعمارهم بين 5 سنوات وأقل في منطقة متوطنة في زيمبابوي ، على مدى سنة واحدة.

الطرق: أجريت هذه الدراسة من شباط 2016 - شباط 2017 في منطقة مادزيوا, مقاطعة شامفا. وبعد إجراء التعبئة للمجتمع المحلي, أحضرت الأمهات أطفالهن البالغين خمسة أعوام وما دون لوضعهم عند خط الأساس وجمع عينات البول, 3و 6و 9 و12 شهرا لمتابعة الفحوصات. في كل مرحلة زمنية, يتم فحص البول من داء البلهارسيا بواسطة ترشيح البول, ويتم إعطاء العلاج للأطفال المصابين. تم تقييم انتشار البلهارسيا الدموية والإصابات بالإضافة لمشاركة الاطفال وتقديم عينة البول في كل زيارة في كل نقطة زمنية لمدة عام واحد.

النتائج:شارك 169 طفل بشكل مستمر في كل الفحوصات وهو ما نسبته (31.6%) من أصل 535 طفل تم اختيارهم من المجتمعات الخمسة. كان أعلى متوسط عدد عينات مسجّل هو 2.9 ضمن المجتمعات ونقاط الدراسة. انخفض انتشار البلهارسيا الدموية بصورة ملحوظة في خط الأساس من 13.3% إلى 2.8% خلال 12 شهرا لحميع المشاركين, ومن 24.9% إلى 1.8% خلال 12 شهرا (P > 0.001) للمشاركين الذين جاءوا في جميع المراحل. بين المجتمعات ، تم العثور على أعلى معدل انتشار خط الأساس في تشيهوري لكلا الفئتين المشاركين الذين قدموا على التوالي (38.5 ٪ ، 26/10) وجميع المشاركين (20.4 ٪ ، 103/21). كانت الإصابات مرتفعة بشكل كبير في 9 شهور فترة متابعة الدراسة (P = 0.021) وفي مابفور (P =0.003). انخفضت الإصابات الجديدة بشكل كبير بمرور الوقت(P< 0.001) أظهر تحليل التراجع اللوجستي أن خطر الاصابة بداء البلهارسيا كان مرتفعا في بعض المجتمعات P< 0.05)

الاستنتاجات:عدوى البلهارسيا الدمويةوالإصابة تكون موسمية وتعتمد على ظروف جغرافية ضئيلة. خطر الاصابة بداء البلهارسيا ضمن أطفال ما قبل المدرسة يرتفع بازدياد العمر. يقلل العلاج المستمر للأفراد المصابين في المجتمع من تفشي المرض لوقت أطول. يعتبر الالتزام بالمشاركة في زيارات مستمرة والإلتزام بتقديم عينة مهماً لتدخلات السيطرة الفعالة.

Translated from English version into Arabic by Free bird, proofread by Abdessalam AIT TOUIJAR, through

TRANSLATORS WITHOUT BORDERS

津巴布韦北部高度流行区学龄前儿童尿道血吸虫病的再感染情况

Masceline Jenipher Mutsaka-Makuvaza, Zvifadzo Matsena-Zingoni, Cremance Tshuma, Sunanda Ray, Xiao-Nong Zhou, Bonnie Webster, Nicholas Midzi

摘要

引言：由于防治目标转变为消除血吸虫病，因此需要干预策略中所有高危年龄组的再感染模式、参与度和样本提交依从性等相关资料数据。本研究旨在评估津巴布韦流行区一年中5岁及以下儿童血吸虫病的流行率、持续参与度、再感染情况、样本提交依从性以及治疗效果。
方法：本研究于2016年2月至2017年2月在沙姆瓦的麦德兹瓦地区进行。在社区动员后，母亲们将其5岁及以下的孩子带来，并分别在基线时和后期随访的第3个月、6个月、9个月和12个月收集儿童尿样。在每个时间点，通过过滤尿液检测尿道血吸虫病，并对阳性者进行治疗。在一年内的每个时间点对埃及血吸虫的流行率、再感染情况，以及儿童参与度和每次随访时提交的尿样进行评估。
结果：5 个社区招募的 535 名儿童中，169 名（31.6%）连续参与全部调查。在社区和调查点中提交样本的最高平均数为 2.9。12 月后，所有参与者的埃及血吸虫病患病率从基线时的 13.3%显著降低至 2.8%，参与全部调查的参与者中患病率从基线的 24.9%显著降低至 1.8%（$P < 0.001$）。在 5 个社区中，Chihuri 的基线患病率在连续参与者（38.5%，10/26）和所有参与者（20.4%，21/103）中均最高。再感染率在随访 9 个月后（$P = 0.021$）和 Mupfure 地区（$P = 0.003$）显著升高。随着时间的推移新发感染显著减低（$P < 0.001$）。Logistic 回归分析显示，某些社区感染血吸虫病的风险较高（$P < 0.05$）。
结论：埃及血吸虫感染和再感染是季节性的，且取决于地理微环境。学龄前儿童感染血吸虫的风险随着年龄的增长而增加。在社区中对感染人群进行持续治疗可降低患病率。连续随访时的参与合规性和样品提交依从性对于控制干预措施的有效性是非常重要的。

Translated from English version into Chinese by Peng Song, edited by Pin Yang

CHINESE CENTER FOR DISEASE CONTROL AND PREVENTION
NATIONAL INSTITUTE OF PARASITIC DISEASES

Ré-infection de la schistosomiase urogénitale chez les enfants d'âge préscolaire dans un district hautement endémique du nord du Zimbabwe

Masceline Jenipher Mutsaka-Makuvaza, Zvifadzo Matsena-Zingoni, Cremance Tshuma, Sunanda Ray, Xiao-Nong Zhou, Bonnie Webster, Nicholas Midzi

Résumé
Contexte: Compte tenu de la tendance à viser l'élimination de la schistosomiase, les éléments suivants sont nécessaires: des données sur les schémas de réinfection, la participation et l'adhésion de tous les groupes d'âge à haut risque aux stratégies d'intervention. Cette étude a été menée pour évaluer la prévalence, les réinfections avec participation consécutive, l'observation de la soumission des échantillons et l'effet du traitement sur la prévalence de la schistosomiase chez les enfants âgés de 5 ans et moins dans un district endémique du Zimbabwe, pendant un an.
Méthodes: L'étude a été menée entre février 2016 et février 2017 dans deux villages de banlieue et deux villages ruraux de la région de Mahenge. Après la mobilisation de la communauté, les mères ont amené leurs enfants âgés de 5 ans et moins pour le recrutement au départ, ainsi que pour le prélèvement d'échantillons d'urine pendant 3, 6, 9 et 12 mois. À chaque moment, l'urine a été testée pour la schistosomiase urogénitale par filtration de l'urine et les enfants ont trouvé un traitement positif. La

prévalence de*Schistosoma haematobium* , les réinfections ainsi que la participation des enfants et la soumission des échantillons d'urine à chaque visite ont été évaluées à chaque moment pendant un an.
Résultats: Sur les 535 enfants recrutés dans les cinq communautés, 169 (31,6%) ont participé consécutivement à tous les points de l'enquête. Le plus grand nombre moyen d'échantillons soumis était de 2,9 entre les communautés et les points d'enquête. La prévalence de *S. haematobium*a diminué de façon significative, passant de 13,3% au départ à 2,8% au bout de 12 mois pour tous les participants et de 24,9% au départ à 1,8% sur 12 mois (P< 0.001) pour les participants ayant répondu à tous les points de l'enquête. Parmi les communautés, la prévalence de base la plus élevée a été observée à Chihuri pour les deux participants venant successivement (38,5%, 10/26) et tous les participants (20,4%, 21/103). Les ré-infections étaient significativement élevées lors de l'enquête de suivi effectuée sur 9 mois (P = 0,021) et à Mupfure(P = 0,003). Les nouvelles infections ont diminué significativement au fil du temps(P < 0,001). Une analyse de régression logistique a montré que le risque de contracter la schistosomiase était élevé dans certaines communautés (P < 0,05).
Conclusions : *Les infections et réinfections dues à S. haematobium* sont saisonnières et dépendent de paramètres micro-géographiques. Le risque d'infection par des schistosomes chez les enfants d'âge préscolaire augmente avec l'âge. Le traitement prolongé des personnes infectées dans une communauté réduit la prévalence au cours du temps. La conformité à la participation lors des visites consécutives et l'adhérence des échantillons sont importantes pour assurer des interventions de contrôle opérationnel efficaces.

Translated from English version into French by William Squire, proofread by Eve Anderson, through

TRANSLATORS
WITHOUT BORDERS

Реинфицирование урогенитальным шистосомозом у детей дошкольного возраста в высокоэндемичном районе в Северном Зимбабве

Маселин Дженифер Мутсака-Макуваза [Masceline Jenipher Mutsaka-Makuvaza], Звифадзо Матсена-Зингони [Zvifadzo Matsena-Zingoni], Креманс Тшума [Cremance Tshuma], Сусанда Рэй [Sunanda Ray], Сяо-Нун Чжоу [Xiao-Nong Zhou], Бонни Вебстер [Bonnie Webster], Николас Мидзи [Nicholas Midzi]

Аннотация

Справочная информация: В связи со смещением цели в направлении искоренения шистосомоза, необходимо следующее: данные о моделях реинфицирования, обеспечение участия и взятия образцов у всех возрастных групп высокого риска с целью осуществления мер по борьбе с заболеванием. Данное исследование проводилось с целью оценки распространения инфекции, уровня реинфицирования, а также последовательного участия и соблюдения режима взятия образцов, а также действенности мероприятий, направленных на сокращение масштабов

распространения шистосомоза у детей в возрасте до 5 лет в эндемичном районе Зимбабве в течение одного года.

Методы: Исследование проводилось с февраля 2016 года по февраль 2017 года в Мадзива района Шамва. В результате мобилизации общин матери привезли своих детей в возрасте 5 лет и моложе для набора на начальном уровне исследования, а также для сбора образцов мочи в начале исследования, через 3, 6, 9 и 12 месяцев. В каждый контрольный момент времени моча тестировалась на урогенитальный шистосомоз путём фильтрования мочи, и дети с положительным результатом получали медицинскую помощь. В течение одного года в каждый момент времени при каждом посещении оценивались распространение возбудителя *Schistosoma haematobium*и реинфицирование, а также участие детей и подача образцов мочи.

Результаты: Из 535 детей, набранных из пяти общин, последовательно на всех стадиях исследования участвовали 169 (31,6%). Среди общин и пунктов исследования наибольшее среднее количество представленных образцов составило 2,9. Уровень распространения возбудителя*S. haematobium* значительно снизился с 13,3% на исходном уровне до 2,8% через 12 месяцев среди всех участников и с 24,9% в начале исследования до 1,8% через 12 месяцев (*P* < 0,001) среди участников, прошедших все стадии исследования в течение всего времени. Среди общин самый высокий уровень распространения на момент включения в исследование был обнаружен в Чихури как среди последовательно участвовавших (38,5%, 10/26), так и среди всех участников (20,4%, 21/103). Уровень реинфицирования был значительно выше в течение 9 месяцев исследования (*P* = 0,021) в районе Мупфуре (*P* =0,003). Уровень новых случаев инфицирования значительно снизился с течением времени (*P* < 0,001). Логистический регрессионный анализ показал, что риск инфицирования шистосомозом был высоким в некоторых сообществах (*P* < 0,05).

Выводы: Инфицирование и реинфицирование возбудителем *S. haematobium* являются сезонными и зависят от микрогеографических условий. Риск инфицирования шистосомами у детей дошкольного возраста повышается с возрастом. Своевременное лечение инфицированных лиц в общине снижает продолжительность сверхурочной работы врачей. Соблюдение последовательности участия и требований к взятию образцов важны для эффективных мер оперативного контроля.

Translated from English version into Russian by Anna Romanenko, proofread by Liudmila Tomanek, through

TRANSLATORS WITHOUT BORDERS

Reinfección de esquistosomiasis urogenital en niños en edad preescolar en un distrito altamente endémico en el norte de Zimbabue

Masceline Jenipher Mutsaka-Makuvaza, Zvifadzo Matsena-Zingoni, Cremance Tshuma, Sunanda Ray, Xiao-Nong Zhou, Bonnie Webster, Nicholas Midzi

Resumen

Antecedentes: A la luz de la tendencia hacia la eliminación de la esquistosomiasis, se requiere lo siguiente: datos sobre los patrones de reinfección, la participación y el cumplimiento de la presentación de muestras de todos los grupos de edad de alto riesgo para las estrategias de intervención. Este estudio se realizó para evaluar la prevalencia, las reinfecciones junto con la participación consecutiva, el cumplimiento de la presentación de muestras, y el efecto del tratamiento sobre la prevalencia de la esquistosomiasis en niños de 5 años o menos en un distrito endémico de Zimbabue, a lo largo de un año.

Metodología: El estudio se realizó entre febrero de 2016 y febrero de 2017 en el área de Madziwa, distrito de Shamva. A partir de la movilización comunitaria, las madres llevaron a sus hijos de 5 años o menos para el reclutamiento al inicio del estudio y también sondeos de sequimiento con la recolección de muestras de orina al inicio, 3, 6, 9 y 12 meses. En cada punto de tiempo, la orina se evaluó para la esquistosomiasis urogenital mediante la filtración de orina y los niños encontraron un tratamiento recibido positivo. *La prevalencia de Schistosoma haematobium,* las reinfecciones, así como la participación de los niños, y la presentación de muestras de orina en cada visita se evaluaron en cada punto de tiempo durante un año.

Resultados: De los 535 niños reclutados de las cinco comunidades, 169 (31.6%) participaron consecutivamente en todos los puntos de la encuesta. El mayor número promedio de muestras enviadas fue de 2.9 entre comunidades y puntos de encuesta. *La prevalencia de S. haematobium,*se redujo significativamente del 13.3% al inicio del estudio al 2.8% a los 12 meses para todos los participantes y del 24.9% al inicio del estudio al 1.8% a los 12 meses ($P < 0.001$) para participantes que acudieron en todos los puntos de tiempo. Entre las comunidades, la mayor prevalencia al inicio del estudio se encontró en Chihuri tanto para los participantes que acudían consecutivamente (38.5%, 10/26) como para todos los participantes (20.4%, 21/103). Las reinfecciones fueron significativamente altas a los 9 meses de la encuesta de seguimiento ($P = 0.021$) y en Mupfure ($P = 0.003$). Nuevas infecciones disminuyeron significativamente con el tiempo ($P < 0.001$). El análisis de regresión logística mostró que el riesgo de adquirir esquistosomiasis era alto en algunas comunidades ($P < 0.05$).

Conclusiones: Las infecciones y reinfecciones por S. haematobium son estacionales y dependen de entornos microgeográficos. El riesgo de infectarse con esquistosomas en niños en edad preescolar se incrementa con la edad. El tratamiento sostenido de individuos infectados en una comunidad reduce la prevalencia con el paso del tiempo. El cumplimiento de la participación en visitas consecutivas y el cumplimiento de la presentación de muestras son importantes para las intervenciones efectivas de control operacional.

Translated from English version into Spanish by Reina X. Sanjurjo, proofread by Manuela Evans, through

TRANSLATORS
WITHOUT BORDERS
